# Effect of Converting Kinematic Aligned Total Knee Arthroplasty to Mechanical Axis Revision Total Knee Arthroplasty on Gap Measurements

**DOI:** 10.7759/cureus.82148

**Published:** 2025-04-12

**Authors:** Spencer J Montgomery, James H Sikes, Drew P Melancon, Humberto A Aparicio, Isaac J Spears, Evan H Powers

**Affiliations:** 1 Orthopaedic Surgery, University of Mississippi Medical Center, Jackson, USA; 2 Medical School, University of Mississippi Medical Center, Jackson, USA

**Keywords:** kinematic alignment, mechanical alignment, revision hip and knee surgery, revision joint replacement, revision knee arthroplasty, revision total knee replacement, total knee arthroplasty technique, total knee replacement (tkr)

## Abstract

Background: Implants and fixation in revision total knee arthroplasty (rTKA) are based on intramedullary referencing and mechanical axis (MA) restoration. Alternative alignment strategies to primary MA total knee arthroplasty (TKA) are increasing in popularity and often place implants in positions of joint line obliquity. The deviation in implant position could result in significant bony defects when being revised to MA-based revision reconstructions. The purpose of this study was to analyze the medial and lateral, as well as flexion and extension gaps, following a standardized workflow to revise a kinematically aligned total knee arthroplasty (KA TKA) to an MA rTKA.

Methods: Seven cadaveric lower extremities that previously underwent caliper-verified KA TKA were converted to MA rTKA utilizing a series of sequential soft tissue releases followed by a tibial osteotomy set perpendicular to the tibial mechanical axis. Gap measurements following each step were recorded using a digital gap-balancing device.

Results: After conversion from KA TKA to MA rTKA, statistically significant increases were observed in the medial extension, medial flexion, lateral extension, and lateral flexion spaces of 1.6 mm (p=0.033), 3. 6mm (p<0.001), 5.6 mm (p<0.001) and 6.9 mm (p<0.001), respectively. Release of the posterior cruciate ligament (PCL) resulted in isolated flexion space opening by 2.4 mm (p=0.002) and 2.3 mm (p=0.022), respectively, for the medial and lateral flexion gaps.

Conclusion: Soft tissue releases seen in rTKA have minimal effect on the medial laxity in extension. In specimens with only mild deviation from neutral alignment and joint line obliquity, the conversion from caliper-verified KA TKA to MA rTKA still resulted in large increases in the lateral-sided gaps, especially in the flexion space. This may create issues with current implant offerings, and surgeons should anticipate substantial augmentation or joint line adjustments when revising implants that were placed with intentional joint line obliquity.

## Introduction

The volume of revision total knee arthroplasty (rTKA) increased substantially in recent years with an increase of 29% cited from 2012 to 2019 [1]. This rise in rTKA procedures can be attributed to an increase in primary TKA as well as an aging population, many of which desire to stay active [2]. Common mechanisms of failure leading to rTKA include infection (19.3%%), aseptic loosening (12.8%), and mechanical complications (7.9%) [2]. Thiele et al. found that implant-associated failure such as polyethylene wear is not as prominent as previously thought, and other studies suggest instability may be the cause for 10-26% of all revision total knee replacements [3,4]. Compared to the innovations seen in primary TKA, limited advancements in rTKA have been made, particularly in the realm of implant alignment.

First described by Insall et al. and long considered the gold standard for total knee arthroplasty (TKA), mechanically aligned total knee arthroplasty (MA TKA) involves perpendicular distal femoral and proximal tibial cuts to the mechanical axis (MA) [5]. However, in contrast to this, Howell et al. presented calipered kinematically aligned total knee arthroplasty (KA TKA), which involves patient-specific bony resections that respect the measured ligament tension to match the implant thickness [6]. This approach aims to use TKA implants to restore the articular surface of the patients’ pre-arthritic joint. Mechanical alignment and kinematic alignment have not shown significant differences in the rates of revision procedures or wear rates in components [7,8]. Some have identified KA TKA as a potentially superior approach to primary TKA with increased patient-reported outcomes, lower pain scores, and maximum flexion compared to MA TKA [7,9]. Furthermore, with the increase in the use of enabling technologies in knee replacement surgery, many other alternative alignment strategies have been proposed that typically incorporate some degree of residual joint line obliquity [10]. These alternative approaches to primary TKA are a deviation from the MA philosophy that formed the basis for rTKA implants and may give rise to more complex surgeries when simultaneously eliminating joint line obliquity and managing relative bony defects due to the bony resections performed at the time of initial surgery [11]. Internal rotation of femoral components following primary KA TKA may result in a larger magnitude of bone removal from the posterior lateral condyle than typically expected, which would give rise to significant challenges when balancing the flexion gap in MA rTKA. There are currently no cadaveric studies that objectively describe the effect of converting a primary KA TKA knee to an MA rTKA on independent gap measurements. The goal of this study is to analyze the impact of standard soft tissue releases and neutralization of the joint line on the medial and lateral, as well as the flexion and extension gaps.

## Materials and methods

Subjects

A cadaveric surgical procedural study was completed. This study was a continuation of a previously conducted cadaveric surgical dissection project, where seven lower extremity cadaveric knees underwent primary KA TKA utilizing imageless, accelerometer-based navigation (Orthalign®, Irvine, CA) with accuracy of the procedure confirmed through caliper verification [12]. The first lower extremity was initially excluded from the first study and was not included in the final analysis as it served to establish the workflow and equipment setup. In total, seven lower extremity knees were included in the analysis: four left knees and three right knees.

All specimens were in the sixth to eighth decades of life with no prior knee surgeries or known injuries to the knee, and all specimens had a BMI<40. Inclusion criteria included male or female specimens with intact bilateral lower extremities. Specimens were required to have no traumatic distortion or previous surgeries of the lower extremities to be included in the study. Exclusion criteria included specimens that did not have intact lower extremities. Inadequate preservation that distorted the anatomy of the lower extremities excluded specimens from the study. All specimens were preserved in a low-temperature environment. No specimens were excluded from the study.

Outcome parameters

Pre-operative full-length lower extremity x-rays were utilized to identify the hip-knee-ankle angle (HKA), mechanical lateral distal femoral angle (mLDFA), and mechanical medial proximal tibial angle (mMPTA) (Table 1). A standard caliper was used to record the thickness of bone fragments in the initial surgery (medial distal femoral, lateral distal femoral, medial posterior femoral, lateral posterior femoral, medial tibial, and lateral tibial). No ligament releases were performed in the initial procedure. The primary outcomes assessed were the changes seen in gap measurements (medial extension, lateral extension, medial flexion, and lateral flexion) following sequential soft tissue releases and finally conversion of the tibial osteotomy to MA orientation utilizing an implant agnostic accelerometer-based navigation system (Table 2).

**Table 1:** Pre-operative specimen measurements. mLDFA, mechanical lateral distal femoral angle; mMPTA, mechanical medial proximal tibial angle; HKA, hip-knee-ankle angle; CPAK, coronal plane alignment of the knee

Specimen Number	1	2	3	4	5	6	7
mLDFA (deg)	88.0	86.5	86.5	84.0	84.0	89.0	88.0
mMPTA (deg)	86.0	86.0	86.0	86.0	86.0	86.0	85.0
Femoral Valgus (deg)	2.0	3.5	3.0	6.0	6.0	1.0	2.0
Femoral Flexion (deg)	3.0	3.5	3.0	4.0	4.0	4.0	4.0
Tibial Varus (deg)	4.0	3.5	4.0	4.0	4.0	4.0	5.0
Tibial Slope (deg)	5.0	5.0	5.0	5.0	5.0	5.0	5.0
Arithmetic HKA (deg)	-2.0	-0.5	-0.5	2.0	2.0	-3.0	-3.0
Joint Line Obliquity (deg)	174.0	172.5	172.5	170.0	170.0	175.0	173.0
CPAK Class	2	2	2	2	2	1	1

**Table 2:** Initial gap measurements and interval changes with each step of the conversion to mechanical alignment. P-value <0.05 in bold indicates a significant change from the previous measurement.

Specimen Number	1	2	3	4	5	6	7	Mean±SD	p-value
Initial Gap Measurement									
Medial Extension Gap (mm)	19	19	19	19	21	20	19	19.43±0.78	
Lateral Extension Gap (mm)	19	19	19	19	21	20	19	19.43±0.78	
Medial Flexion Gap (mm)	20	19	19	19	21	20	21	19.86±0.89	
Lateral Flexion Gap (mm)	20	19	23	21	23	24	21	21.57±1.81	
After Medial Release									
Medial Extension Gap (mm)	2	0	2	0	1	0	0	0.71±0.95	0.094
Lateral Extension Gap (mm)	0	0	2	2	3	0	0	1.00 ±1.29	0.086
Medial Flexion Gap (mm)	1	2	2	0	1	-1	0	0.71±1.11	0.140
Lateral Flexion Gap (mm)	-1	2	3	0	1	-1	2	0.86±1.57	0.200
After the Box Cut and Posterior Collateral Ligament Resection									
Medial Extension Gap (mm)	1	0	-1	0	0	0	0	0.00±0.57	1.000
Lateral Extension Gap (mm)	1	0	-1	0	0	0	0	0.00±0.57	1.000
Medial Flexion Gap (mm)	2	4	1	2	3	3	2	2.43±0.97	0.002
Lateral Flexion Gap (mm)	2	4	0	0	3	3	4	2.29±1.70	0.022
After Posterior Capsule Resection									
Medial Extension Gap (mm)	0	0	1	0	0	0	0	0.14±0.37	0.356
Lateral Extension Gap (mm)	0	0	1	0	0	0	0	0.14±0.37	0.356
Medial Flexion Gap (mm)	1	-1	0	1	0	0	1	0.29±0.75	0.356
Lateral Flexion Gap (mm)	1	-1	0	1	0	0	1	0.29±0.75	0.356
After Converting to Mechanically Aligned Tibia									
Medial Extension Gap (mm)	1	1	1	0	0	0	2	0.71±0.75	0.047
Lateral Extension Gap (mm)	3	5	5	4	2	4	8	4.42±1.90	<0.001
Medial Flexion Gap (mm)	0	0	0	1	0	0	0	0.14±0.37	0.356
Lateral Flexion Gap (mm)	5	4	4	3	2	2	4	3.28±0.95	<0.001
Total Change in Gap Measurement									
Medial Extension Gap (mm)	4	1	3	0	1	0	2	1.57±1.51	0.033
Lateral Extension Gap (mm)	4	5	7	6	5	4	8	5.57±1.51	<0.001
Medial Flexion Gap (mm)	4	5	3	4	4	2	3	3.57±0.98	<0.001
Lateral Flexion Gap (mm)	7	9	7	4	6	4	11	6.86±2.54	<0.001

Gap balancing and measurement

**Figure 1:**
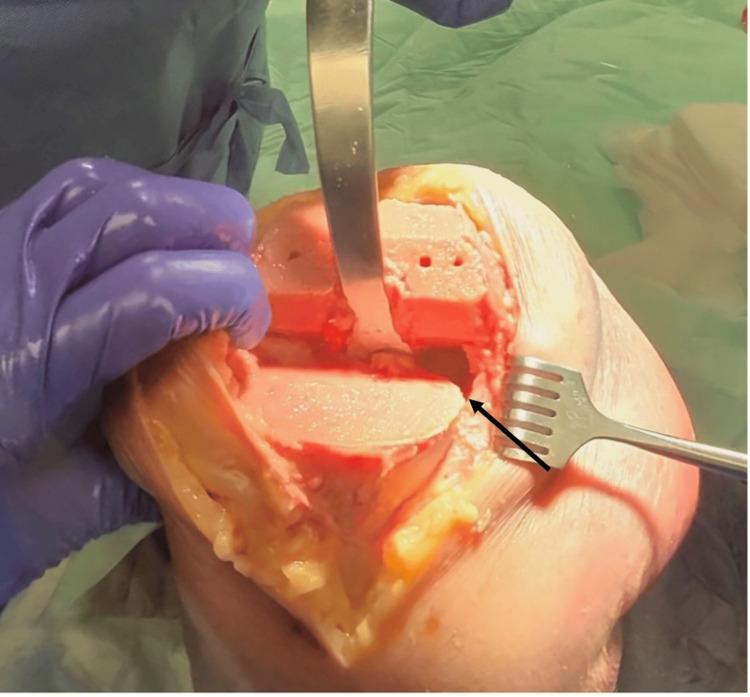
Demonstration of medial release, ensuring 1.5 cm of clearance distally from the osteotomy surface, from the tibial tubercle to the most posterior aspect of the medial tibial plateau.

All procedures were completed by the senior author, and all data was collected over a four-week period. Before proceeding, a physical examination of all specimens by the senior author was completed to confirm the physiological laxities of the knees used in this study. Prior to the steps discussed in this analysis, each lower extremity underwent a primary TKA using a standard medial parapatellar approach with a longitudinal quadriceps muscles cut, utilizing an imageless, accelerometer-based navigation unit adhering to kinematic alignment principles. Bone removal was accurately performed with a caliper-verified kinematic alignment technique. In the initial operation, radiographic measurements of mMPTA and mLDFA were utilized, with femoral flexion set to 4 degrees and tibial slope set to 5 degrees for all specimens. Bone fragments were measured with a caliper following resections to confirm thickness; there were no ligament releases or soft tissue balancing measures performed. The conversion from KA TKA to MA rTKA began by obtaining an initial gap measurement. The lower extremity was placed in extension and the paddles of the gap measurement device were inserted into the joint space. The proximal paddle of the gap measurement device rotates about a central axis to provide independent medial and lateral measurements when resting against the femoral condyles. A torque driver was inserted and rotated clockwise until a click was heard and felt. The tension applied with this device was limited to 30 lbs per the torque driver handle, which applies between 250 N and 300 N to the joint space (OrthAlign, data on file). The medial and lateral extension gaps were recorded. The lower extremity was then placed in 90 degrees of flexion, and the same process was repeated to obtain the initial medial and lateral flexion gaps. Soft tissue releases began with a medial release, which involved clearing the medial proximal tibia from the tibial tubercle to the most posterior aspect of the medial tibial condyle, extending 1.5 cm below the joint line (Figure 1).

**Figure 2:**
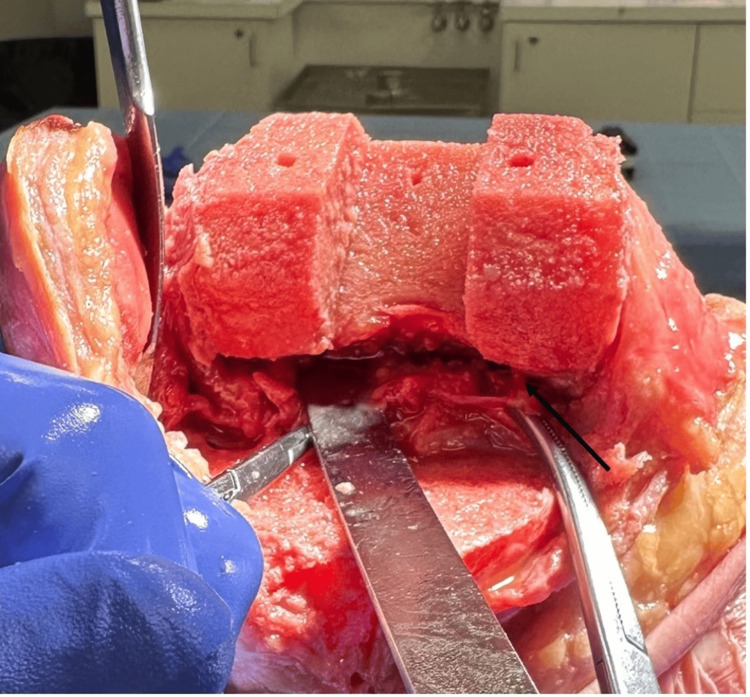
Demonstration of lateral and medial posterior capsule release (arrow) after box cut preparation and PCL resection. PCL, posterior cruciate ligament

Flexion and extension gaps following the medial releases were obtained using the digital gap balancing device and recorded. The surgeon proceeded with the release of the posterior cruciate ligament (PCL) through a box cut by securing the box-cutting jig on the distal femur. Once the box cut osteotomy was performed, the transected box fragment was placed under tension and the PCL was released. The flexion and extension gaps were then recorded. Next, a scalpel, osteotome, and mallet were used to perform the posterior capsule resection to clear 1.5 cm proximally from the posterior femur superior to the posterior femoral condyle resections (Figure 2). Flexion and extension gaps were recorded.

**Figure 3:**
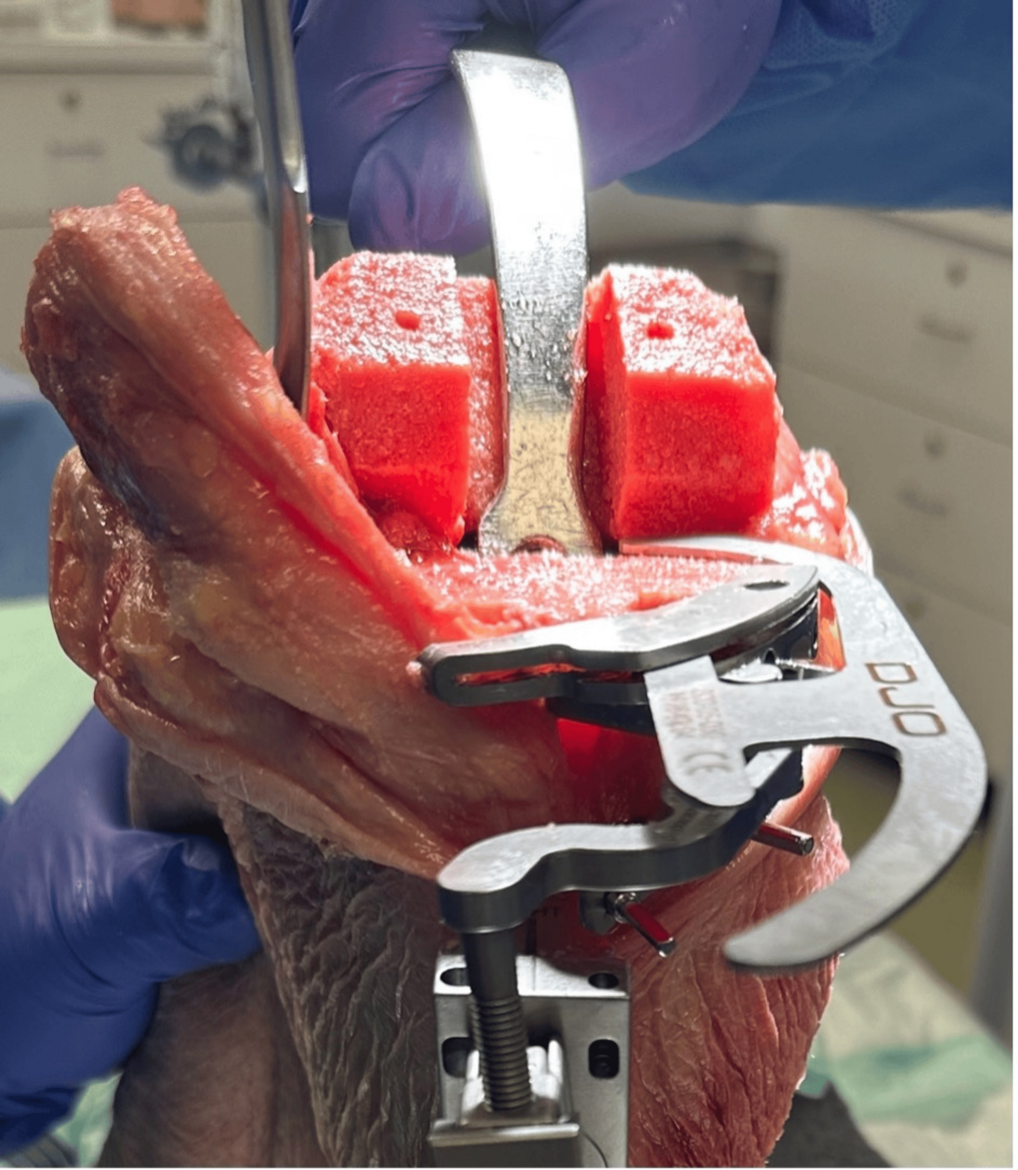
Setting tibial guide depth to posteromedial aspect of tibia.

The final step was conversion to the MA by making a tibial cut perpendicular to the MA. The MA was identified using the accelerometer-based navigation platform. A tibial cutting guide was secured to the proximal tibia and the resection was made at a position of 0o varus and 0o posterior slope; the depth was set to be flush with the lowest portion of the tibial surface (Figure 3). The flexion and extension gaps were recorded for the final time. To minimize confounding from the variable thickness of the bony resection, we reduced all gap measurements universally by the smallest change observed. No revision prosthesis was implemented in this analysis as the goal was strictly to assess gap changes following conversion to MA rTKA.

Statistical analysis

SPSS version 29.0.2.0 (20) (IBM Corp, Armonk, USA) was used to conduct our statistical analysis. All pre-operative data regarding the status of the joint was recorded (Table 1). The mean and standard deviation for the change observed in the medial and lateral flexion and extension gaps were recorded (Table 2). A paired samples T-test was used to determine the significance of the changes in the gap measurements following the interventions using p<0.05 (Table 2). The effect size for the combined soft tissue changes was calculated using Cohen’s d, comparing the mean difference divided by the standard deviation (Table 3). Boxplots were obtained to visualize changes in the gap measurement. Median bars are indicated in the boxplots. Data is stated as mean (SD) or mean, SD.

**Table 3:** Paired t-test and effect size for soft tissue releases in the conversion from KA TKA to MA rTKA. t, t-statistic; df, degrees of freedom

	t	df	p	Cohen’s d	95% Confidence Interval	
					Lower	Upper
Medial Extension Gap	2.750	6	0.033	1.039	0.077	1.951
Lateral Extension Gap	9.750	6	<0.001	3.685	1.520	5.834
Medial Flexion Gap	9.682	6	<0.001	3.660	1.507	5.795
Lateral Flexion Gap	7.129	6	<0.001	2.695	1.021	4.337

## Results

Pre-operative radiographic measurements and resection values from the primary TKA are presented in Table 1. Joint phenotypes were identified using the coronal plane alignment of the knee (CPAK) for purposes of the primary kinematically aligned joint.

After KA TKA utilizing navigation, the average medial extension gap was 19.4 mm (SD 0.78 mm), lateral extension gap was 19.4 mm (SD 0.78 mm), medial flexion gap was 19.9 mm (SD 0.89 mm), and lateral flexion gap was 21.6 mm (SD 1.8 mm), prior to any soft tissue releases or resections, as recorded in Table 2. The change in the flexion and extension gaps following each release is described in Table 2 with the values recorded as the whole number change from the prior measurement.

Medial releases along with the posterior capsule resection resulted in statistically insignificant changes in the gap measurements. Release of the PCL resulted in flexion space opening by 2.4 mm and 2.3 mm, respectively, for the medial and lateral flexion gaps. The final gaps were determined by combining each sequential measurement. Final gap measurements following conversion to the MA were as follows: medial extension gap 21.0 mm (SD 1.4), lateral extension gap 25.0 mm (SD 1.4), medial flexion gap 23.4 mm (SD 1.1), and lateral flexion gap 28.3 mm (SD 2.4).

**Figure 4:**
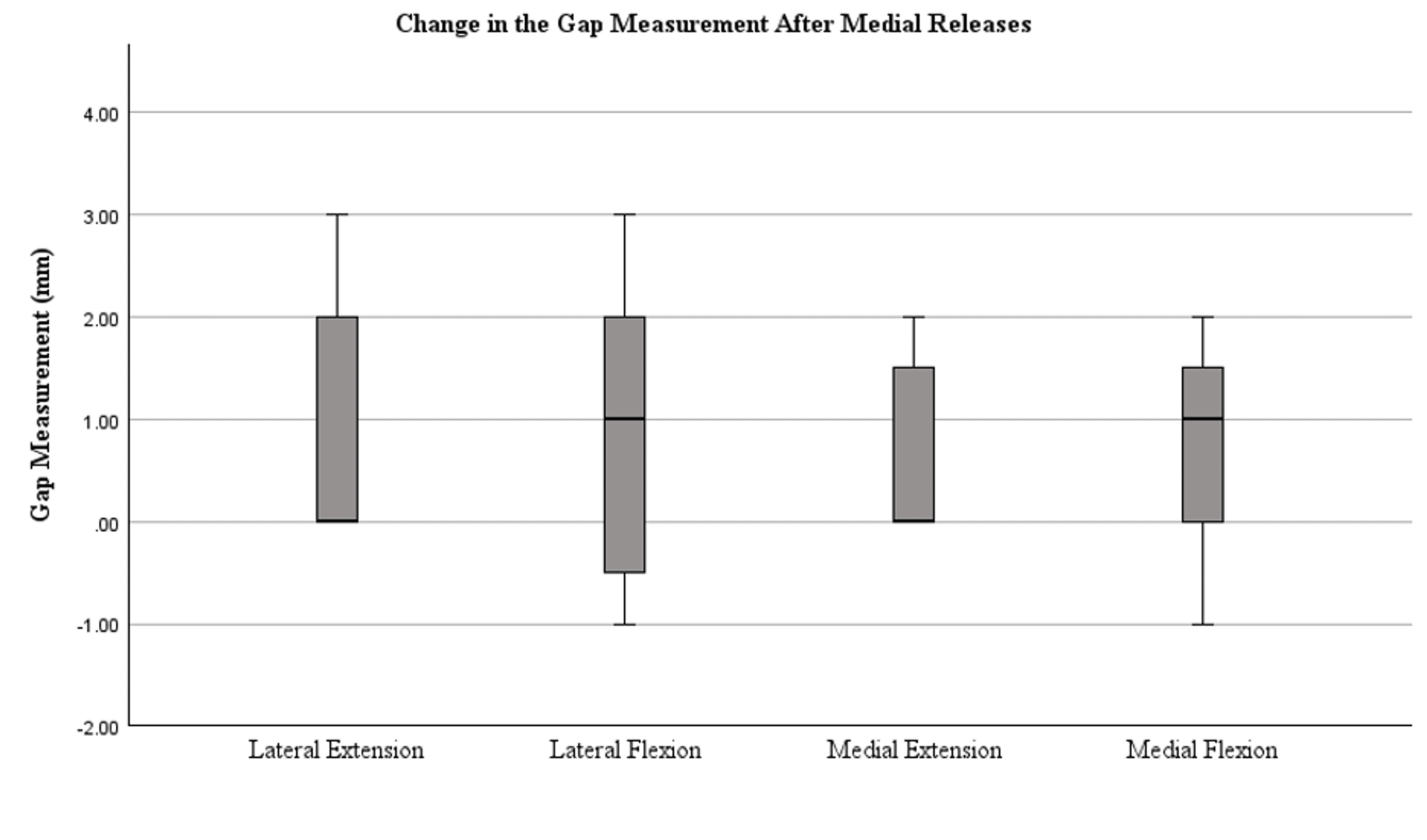
Tibiofemoral joint gaps after medial releases were completed. The presence of an asterisk (*) identifies a significant change in the gap measurements after medial release compared to measurements after KA TKA was performed, defined by a p-value of <0.05. Median bars (bold) are shown within the boxplot. The boxes represent the IQR ranging from the first quartile (25th percentile) to the third quartile (75th percentile). Error bars are plotted based on IQR X 1.5. Values beyond this range are considered outliers. Whiskers of the error bars are omitted if the IQR is 0 where there are too few unique data points. IQR, interquartile range

**Figure 5:**
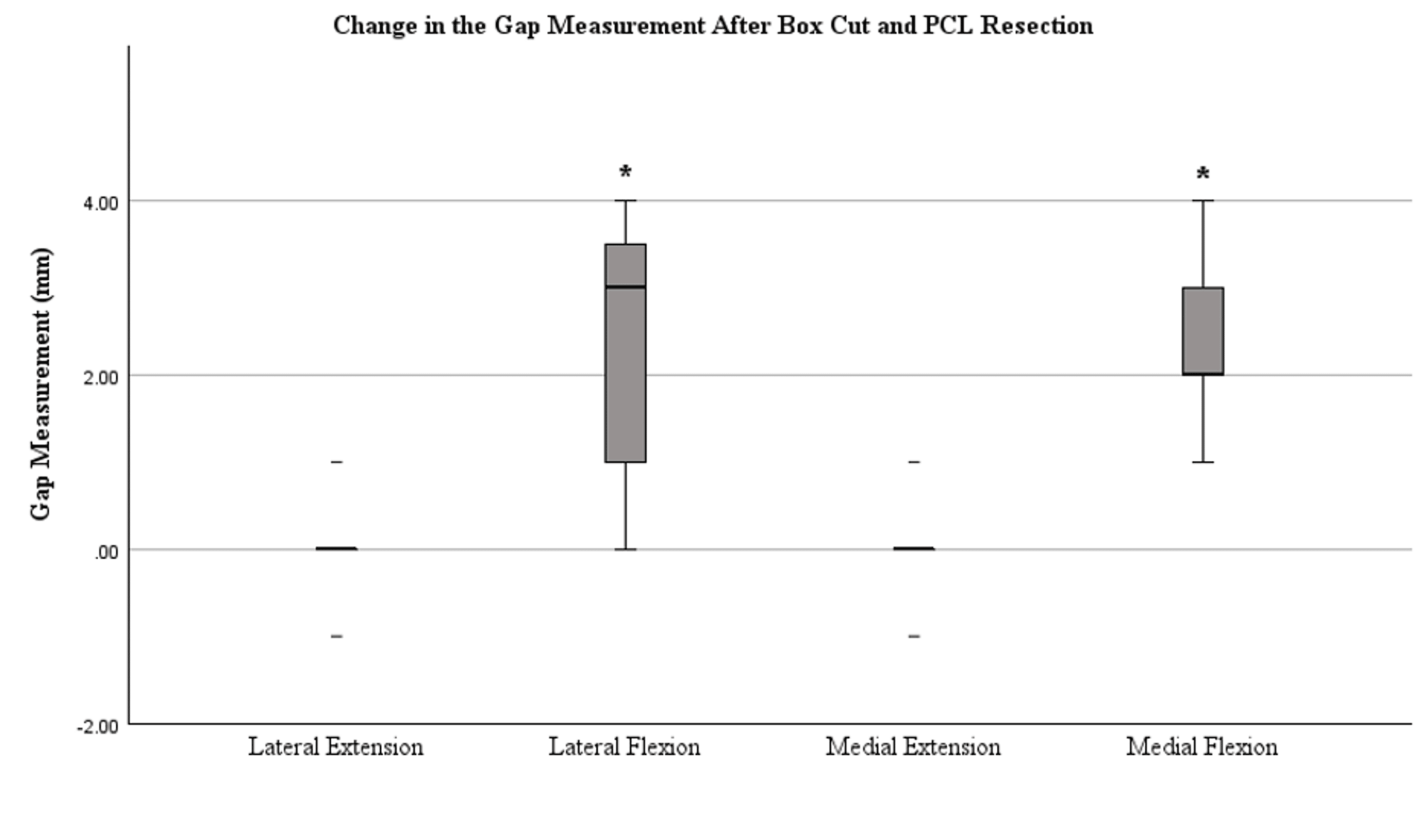
Tibiofemoral joint gaps after the box cut and PCL resection were completed. The presence of an asterisk (*) identifies a significant change in the gap measurements after the box cut and PCL resection were completed compared to measurements after the medial release was performed, defined by a p-value of <0.05. Median bars (bold) are shown within the boxplot. The boxes represent the IQR ranging from the first quartile (25th percentile) to the third quartile (75th percentile). Error bars are plotted based on IQR X 1.5. Values beyond this range are considered outliers. Whiskers of the error bars are omitted if the IQR is 0 where there are too few unique data points. IQR, interquartile range; PCL, posterior cruciate ligament

**Figure 6:**
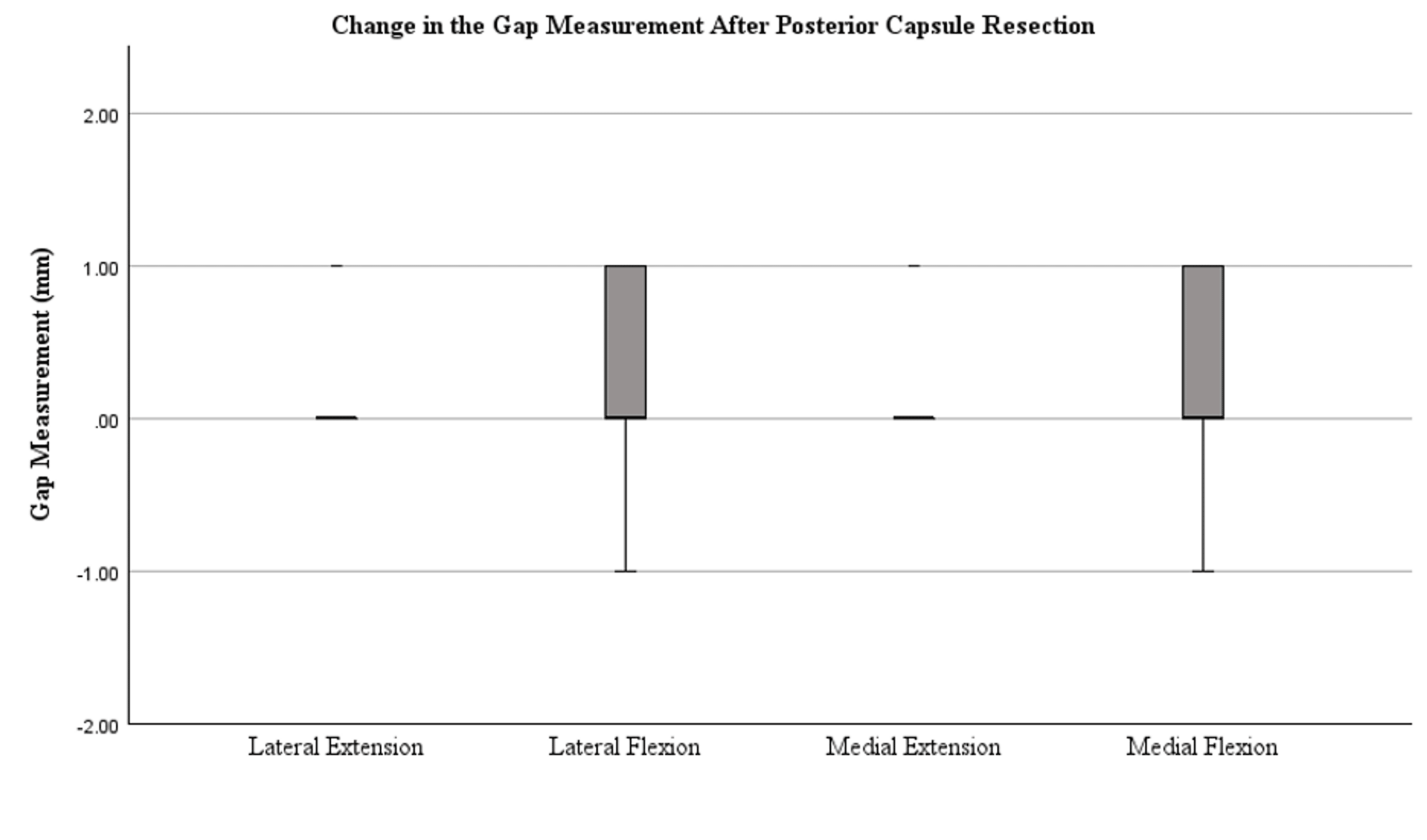
Tibiofemoral joint gaps after the posterior capsule resection was completed. The presence of an asterisk (*) identifies a significant change in the gap measurements after posterior capsule resection compared to measurements after the box cut and PCL resection were completed, defined by a p-value of <0.05. Median bars (bold) are shown within the boxplot. The boxes represent the IQR ranging from the first quartile (25th percentile) to the third quartile (75th percentile). Error bars are plotted based on IQR X 1.5. Values beyond this range are considered outliers. Whiskers of the error bars are omitted if the IQR is 0 where there are too few unique data points. IQR, interquartile range

**Figure 7:**
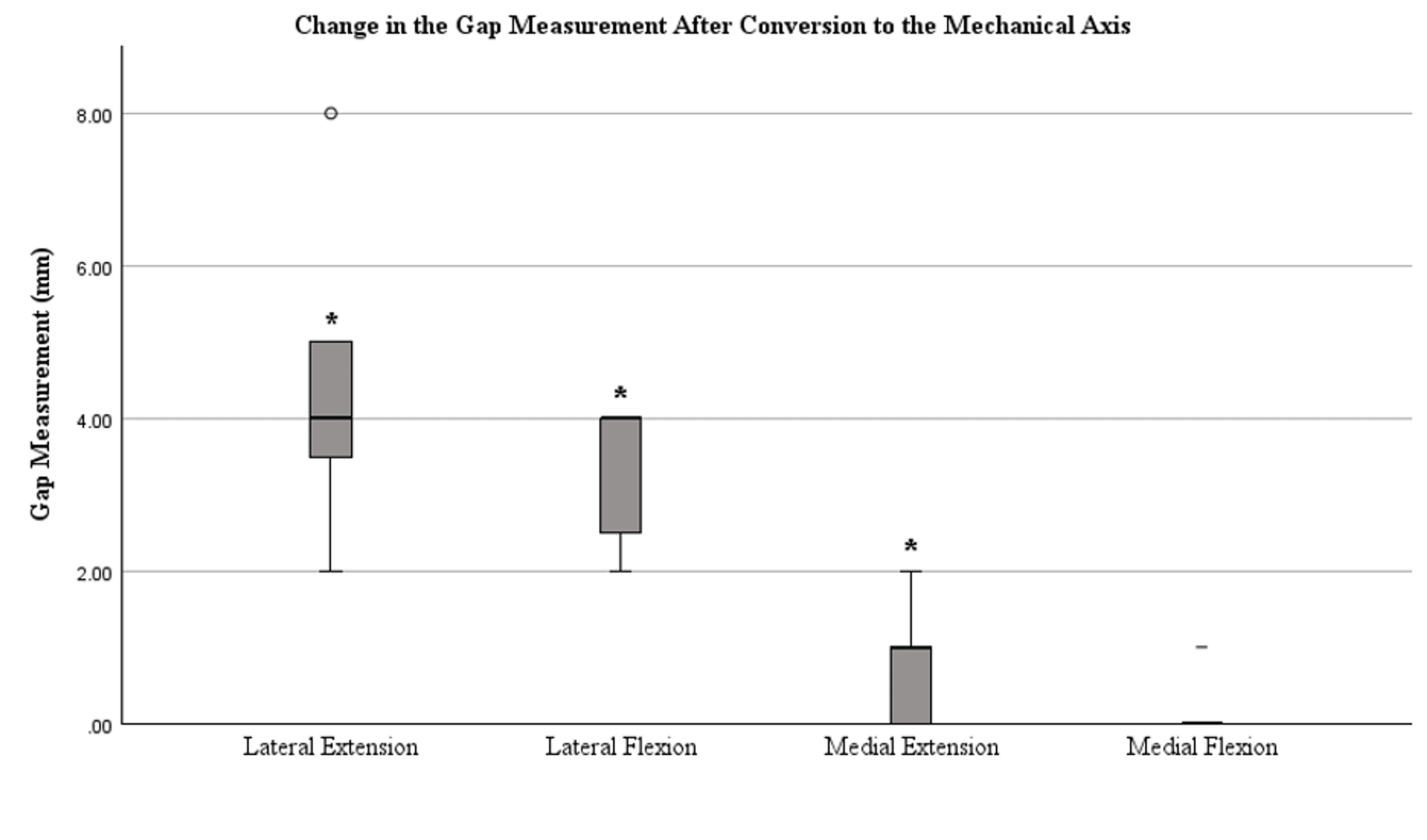
Tibiofemoral joint gaps after conversion to a mechanically aligned tibia. The presence of an asterisk (*) identifies a significant change in the gap measurements after the conversion of proximal tibial osteotomy to navigated neutral orientation compared to measurements after the posterior capsule release was performed, defined by a p-value of <0.05. Outliers are represented by a circle (〇). Median bars (bold) are shown within the boxplot. The boxes represent the IQR ranging from the first quartile (25th percentile) to the third quartile (75th percentile). Error bars are plotted based on IQR X 1.5. Values beyond this range are considered outliers. Whiskers of the error bars are omitted if the IQR is 0 where there are too few unique data points. IQR, interquartile range

Figures 4-7 display the changes in the gaps observed after each release. Significant changes in the gaps were observed in the medial (p=0.002) and lateral (p=0.022) flexion gaps following the release of the PCL. Significant increases in the gaps following tibial conversion to the MA were observed in the lateral extension (p=<0.001), lateral flexion (p=<0.001), and medial extension (p=0.047). The boxes in Figures 4-7 represent the interquartile range (IQR), which is the middle 50% of data distribution, ranging from the first quartile to the third quartile.

## Discussion

This study sought to recreate the in vivo environment during rTKA, including the typical soft tissue releases required for implant exposure necessary for removal, and objectively measure the effects on gap measurements. The results demonstrate substantial increases, particularly in the lateral gap measurements when converting caliper-verified KA TKA knees to MA rTKA in cases with mild deviation from neutral alignment. The CPAK classification is based on the arithmetic HKA in long-leg radiographs, and this classification was used in this study to identify and separate different deformities of the knee. The CPAK classifications stratify knee phenotypes into types 1-9 with the most common deformities being CPAK types 1, 2, and 5 [13]. All specimens in this study had mild varus joint lines (five CPAK 2 and two CPAK 1). Medial releases have traditionally followed a sequential pattern based on the degree of deformity although an agreed-upon sequence and magnitude of medial releases is less clear [14,15]. In this study, the medial release performed included the deep MCL, the posteromedial capsule, the posterior oblique ligament, and potentially elements of the semimembranosus and medial gastrocnemius muscle. We found all gaps opened slightly with the medial releases on average by 0.71 mm (medial extension), 1.0 mm (lateral extension), 0.71 mm (medial flexion), and 0.86 mm (lateral flexion). The increase observed in the medial gap is consistent with previous studies; however, the increase in the lateral gap is discordant with current literature [16].

In this study, PCL resection was performed as part of the femoral box cut and produced an isolated increase in the flexion gap. The medial flexion gap increased by an average of 2.4 mm (SD 0.97 mm), and the lateral flexion gap increased by 2.3 mm (SD 1.7 mm). Foge et al. similarly found increases in the flexion gap after the release of the PCL in cadaveric specimens, although the increases were significantly larger, with a medial flexion gap increase of 3.9 mm to 5.1 mm and a lateral flexion gap increase of 4.2 mm to 4.7 mm [17]. Another study by Kayani et al., which analyzed PCL resection in patients with symptomatic osteoarthritis, found increases more similar to our findings, with a medial flexion gap increase of 2.4 mm (SD 1.5 mm) and a lateral flexion gap increase of 3.3 mm (SD 1.6 mm) [18]. Further supporting our findings, modern techniques with advanced navigation platforms have also shown that the increase in center point joint space after PCL release is closer to 2 mm [19].

Key changes

Posterior capsule resections are generally implemented to correct flexion contracture and thereby increase the degrees of extension [20]. Kinoshita et al. have proposed a “stepwise” release of the posterior capsule in posterior stabilized TKA [20]. In our study, the flexion and extension gaps both saw statistically insignificant, minimal changes following posterior capsule resection in this study. The medial flexion gap increased on average by 0.29 mm (SD 0.75), lateral flexion gap 0.29mm (SD 0.75), medial extension gap 0.14mm (SD 0.37), and lateral extension gap 0.14mm (SD 0.37). Minimal changes in the gap measurements after posterior capsulotomy is contrary to other studies such as that by Kawasaki et al. who found an increase in the extension gap of 1.5 ± 0.73mm [21] and Katagiri et al. who found increases in the medial and lateral extension gaps of 1.3 ± 1.0mm and 2.2 ± 1.5mm respectively, which differs from the minimal increases in the extension gaps observed in our study [22]. Our capsular release followed a similar protocol to Katagiri et al. and we ensured a 1.5 cm release of tissues medially, laterally, and throughout the intercondylar notch, though we analyzed cadaveric tissues while Katagiri et al. analyzed gaps in living patients.

After the bony osteotomy converting the tibia to MA using the navigation device, the combined effects of rTKA in KA knees could truly be appreciated. To minimize confounding from the variable thickness of the bony resection, we reduced all measurements by the smallest change, which, in all but one specimen, was the medial flexion gap. We found that the osteotomy resulted in a further 3.7 mm increase in the lateral compartment in extension compared to the medial side, and a 3.1 mm increase in the lateral compartment in flexion compared to the medial side. In summary, the total gaps produced by converting this series of KA TKA to MA rTKA in medial flexion and extension, and lateral flexion and extension, were 23.4 mm (SD 1.1), 21.0 mm (SD 1.4), 28.3 mm (SD 2.4), and 25.0 mm (SD 1.4), respectively. While previous studies confirm that the physiological flexion and extension gaps are trapezoidal and asymmetric, especially in flexion, this is to a much smaller degree [12,23]. Appropriate knee laxity is a nuanced subject and may differ based on the status of the joint prior to revision and the approach used. Nixon et al. found that patients with 3-6 mm more laxity on the lateral side in flexion experienced better patient-reported outcomes [24]. This was similar to the findings by Okamoto et al., who found 2-5 mm of laxity to be acceptable, and >5 mm of lateral laxity in flexion associated with worse patient-reported outcome scores [25]. The clinical implications of the flexion laxity observed in this analysis may lead to persistent instability and abnormal translational movement of the tibial component in the anterior-posterior plane. Functionally, patients may experience difficulties traversing inclines or declines.

Revision TKA poses an increased technical challenge compared to primary TKA due to joint line recreation, as evidenced by Buller et al., who found a mean joint line elevation of 7.2 ± 6.6 mm postoperatively after rTKA [26]. In addition to joint line positioning, mismatched flexion, and extension gaps require additional balancing and considerations. In attempts to address this, Baldini et al. found that offset stems can be used to account for joint line elevation, and furthermore, this can optimize implant alignment along the MA [27]. Importantly, Wells and Purcell validated that accelerometer-based navigation (Orthalign) can be an asset in rTKA, restoring the joint line and ensuring adequate stability [28]. Furthermore, Patel et al. analyzed the use of Orthalign in patients undergoing primary TKA and found that 96.0% of tibial components and 95.8% of femoral components were within 2 degrees of the target [29]. The reproducibility of the accuracy of the digital gap balancing device was illustrated in the first phase of the dissection. A review of the use of robotic platforms in rTKA suggested the potential benefit of such navigation, but there is still limited evidence regarding whether robot-assisted surgery can improve long-term outcomes [30]. Regardless of the technology utilized, a concern remains that a revision from a previous KA TKA to MA rTKA has a high likelihood of producing a large lateral flexion gap. If the goal were to produce equal and symmetric flexion and extension gaps, or even to retain slight lateral laxity, this gap would require significant augmentation of the femoral component or raising the joint line.

There are several limitations in our study. The sample size and variability of alignment profiles are small. Despite the small sample size, cadaveric studies investigating the biomechanical properties of knee arthroplasty commonly utilize small sample sizes, particularly when assessing aspects of a single approach [16,17]. All the specimens had minimal deviation, with aHKA ranging from 2° valgus to 3° varus. Despite these small degrees of deviation, we still found significant concerns with gaps after conversion to MA rTKA and would expect this to be even more pronounced in CPAK 1 patients and those with more severe mMPTA values. Future studies could include joints with more severe limb malalignment to confirm this. Inherent cadaveric factors, such as inadequate tissue hydration, variable changes in elasticity, and the lack of normal physiological responses like inflammation, may limit the generalizability of the findings. Furthermore, inadequate preservation of specimens could contribute to an abnormal soft tissue environment, potentially preventing accurate measurement of gaps. There may also be limitations to cadaveric studies when assessing ligamentous and soft tissue laxities. Similarly, repeated testing of joints with the tensioning gap measurement device could stretch the supporting ligaments, leading to overestimation of the gaps, although in some cases, repeated testing produced the same or even 1 mm reduced gaps compared to the previous measurement.

## Conclusions

In conclusion, our study showed that the process of exposing the implants for removal during the revision of KA TKA to MA rTKA can produce substantial and predictable increases in extension and flexion gaps. In addition, the bony osteotomy required to revise KA TKA to MA rTKA in CPAK types 1 and 2 produces further iatrogenic laxity, primarily in the lateral compartment, especially affecting the posterior condyle and the subsequent lateral flexion gap. While KA TKA is gaining popularity and building supportive clinical data, it does not prevent the need for rTKA. Based on the findings in this paper, performing a balanced MA rTKA after KA TKA may require substantial augmentation of the lateral flexion gap, leading to challenges in managing flexion and extension mismatch. Future research should investigate the use of robotics in the conversion from KA TKA to MA rTKA or perhaps primary restricted KA, both of which may decrease the amount of rotation; however, the mild deformity in this series would be expected to have no restrictions applied. Advanced technology, additional augmentation options, or specific revision KA implant designs may provide alternative approaches to these challenges. Further research is warranted into the effects of revising more severe alignment variations treated with KA TKA to MA rTKA.
